# Torque–angle relationships of human toe flexor muscles highlight their capacity for propulsion in gait

**DOI:** 10.1242/jeb.249816

**Published:** 2025-01-10

**Authors:** Samuel J. Wisdish, Hannah M. Rice, Dominic J. Farris

**Affiliations:** ^1^ Public Health and Sport Sciences, Faculty of Health and Life Sciences, University of Exeter, St Luke's Campus, Exeter, EX1 2LU, UK; ^2^ Department of Physical Performance, Norwegian School of Sport Sciences, Oslo 0806, Norway

**Keywords:** Biomechanics, Foot, Force, Nerve stimulation, Plantar intrinsic muscles, Walking

## Abstract

Human proficiency for bipedal locomotion relies on the structure and function of our feet, including the interplay between active muscles and passive structures acting on the toes during the propulsive phase of gait. However, our understanding of the relative contributions of these different structures remains incomplete. We aimed to determine the distinct toe-flexion torque–angle relationships of the plantar intrinsic muscles (PIMs), extrinsic muscles and passive structures, therefore offering insight into their force-generating capabilities and importance for walking and running. Torque–angle data were twice collected from nine healthy individuals (6 males, 3 females; 28±5 years) using supramaximal transcutaneous electrical stimuli applied at two tibial nerve sites to distinguish between muscle-driven and passive toe-flexion torque about the metatarsophalangeal (MTP) joint. Innervating extrinsic muscles and PIMs concurrently produced peak torques (hallux=3.05±0.70 N m, MTP angle=48.0±13.6 deg; lesser digits=3.19±0.98 N m, MTP angle=42.6±13.4 deg) exceeding by 208% (hallux) and 150% (lesser digits), respectively, those from PIM stimulation alone. Notably, MTP joint angles pertinent to gait corresponded to the ascending limb of the active torque–angle relationship, with active muscle joint torques being the dominant contributor over passive torques. The latter finding suggests that human toe flexors are well adapted to generate the MTP joint torques that are necessary for walking and running. This further supports the notion that muscles acting within the foot play an important role in the foot's mechanical function and our ability to walk and run in an upright posture.

## Teaser

**Summary:** A systematic evaluation of the torque–angle relationships of the human foot muscles supports the importance of these muscles for healthy foot function and bipedal gait.

## INTRODUCTION

The structure and function of human feet are distinct from those of non-human apes and are considered key indicators of our evolved specialism for bipedalism. Human feet have been considered ‘versatile machines’ (Takahashi et al., 2022) serving as the primary interface between the body and the ground that adjust their stiffness (Farris et al., 2019, 2020) and energetic behaviour (Riddick et al., 2019; Smith et al., 2021; Takahashi et al., 2017) to meet the requirements of everyday activities such as walking and running (Holowka et al., 2021). Their unique structural features, including prominent arches (Davis and Challis, 2023; Venkadesan et al., 2020; Welte et al., 2023), a robust calcaneus (DeSilva et al., 2019), an elastic plantar aponeurosis (PA) (Ker et al., 1987; Stearne et al., 2016) and network of muscles (Farris et al., 2019, 2020; Kelly et al., 2014, 2019; Riddick et al., 2019; Smith et al., 2022a,b), each provide mechanisms to cope with the specific demands of bipedal locomotion. Notably, the active neuromechanical contribution of the plantar intrinsic muscles (PIMs) plays a significant role in the foot's mechanical versatility, possibly outweighing the foot's passive mechanisms (Farris et al., 2019), with greater muscular activation associated with greater mechanical demands (Farris et al., 2020; Kelly et al., 2014). This important function of the plantar intrinsic foot muscles for human gait is supported by comparative work that noted the relatively larger size of some intrinsic toe flexors in humans compared with those of several species of great apes (Oishi et al., 2018; Vereecke et al., 2005) (Table S1 and Fig. S1), suggesting that human foot muscles may have adapted to assist in enabling bipedal gaits.

**List of symbols and abbreviations**

HFT: hallux flexion torque
ICC: intraclass correlation coefficient
*k*: coefficient of stiffness of exponential passive torque-angle curve
LDFT: lesser digits flexion torque
MDC_95%_: minimum detectable change with a 95% confidence interval
MM: medial malleolus stimulation site
MTP: metatarsophalangeal
PA: plantar aponeurosis
PF: popliteal fossa stimulation site
PIMs: plantar intrinsic muscles
SEM: standard error of measurement
*T*_max_: maximum torque
θ_opt_: optimal MTP joint angle, at which *T*_max_ is achieved
θ_slack_: MTP joint slack angle (the greatest MTP joint angle corresponding to 0 N m of passive torque)

The importance of the PIMs becomes notable in their absence. Weakness or atrophy of foot muscles is associated with an increased risk of falling and reduced mobility in older people (Menz et al., 2005, 2006). Furthermore, experiments administering anaesthetic neural blocks to inhibit foot muscle function describe detrimental effects on power production during the propulsive phase of gait (Farris et al., 2019) and vertical jumping (Smith et al., 2022b). Farris et al. (2019) showed that removing the active contribution of the PIMs via anaesthetic nerve block reduced the ability of participants to stiffen the metatarsophalangeal (MTP) joint during late-stance push-off in walking and running. This resulted in compensatory increased hip joint work to maintain locomotor speed. Thus, it is evident that healthy humans use the PIMs' contribution to facilitate bipedal locomotion. Consequently, the force-generating capacity of the toe flexor muscles can be considered a key component of healthy foot function and human ability to walk and run in an upright posture. However, the specific force-producing capabilities of different foot muscle groups (i.e. intrinsic versus extrinsic; hallucal versus lesser digits), and how they compare to passive tissues, remains poorly understood. Therefore, firm mechanistic links between the structure and function of these muscles cannot currently be drawn.

A muscle's ability to produce force *in vivo* can be largely characterised by its torque–angle relationship, analogous to the force–length relationship (Grieve, 1978). In particular, the joint positions at which a muscle most effectively generates torque are indicative of the positions it is optimised for functioning at (Rassier et al., 1999). If a muscle produces greatest torque at the same joint positions that it is active during a movement, we postulate that it is well adapted to help perform that movement or function. However, establishing these torque–angle relationships is challenging because of the foot's complex structure, its multiplicity of muscles and ligaments (including the PA) (Soysa et al., 2012) and a common inability to maximally voluntarily contract individual foot muscles (Olivera et al., 2020). Toe flexion is achieved through activation of the PIMs and extrinsic foot muscles spanning the foot's plantar surface and crossing the MTP joints, with the extrinsic muscles also traversing the ankle joint. As a consequence, toe flexion torque depends on the angular position of both the MTP and ankle joints (Goldmann and Brüggemann, 2012) along with neural activation of the muscle tissue (Olivera et al., 2020). However, individuals struggle to volitionally contract specific foot muscles, such as abductor hallucis, with voluntary contractions resulting in activation deficits of up to 50% compared with contractions evoked through electrical impulses (Olivera et al., 2020). Therefore, although some authors have assessed the strength of foot muscle groups via maximal voluntary efforts (Goldmann and Brüggemann, 2012; Koyama et al., 2019; Kurihara et al., 2014; Yamauchi and Koyama, 2019), this may not accurately identify their true capacity. To better understand the functional capacity of different foot muscles for enabling human locomotion, recently developed experimental protocols for mapping torque–angle relationships of human muscles *in vivo* via peripheral nerve stimulation (Hoffman et al., 2012, 2014; Pincheira et al., 2018) could be employed.

To fully interpret foot muscle function, we must also consider that individual foot muscles follow distinct anatomical pathways, resulting in variation between the actions of toe flexors within the PIMs and extrinsic muscle groups. For example, abductor hallucis and flexor digitorum brevis, the two largest PIMs (Kelly et al., 2014; Kura et al., 1997), attach on separate toes, with the former flexing and abducting the hallux, whilst the latter flexes the lesser digits about the MTP joints. Similarly, the extrinsic foot muscles, flexor hallucis longus and flexor digitorum longus, also act upon the hallux and lesser digits, respectively. Previous voluntary toe flexion strength measurements have often combined the toes as a single segment, integrating contributions from hallucal flexors and lesser digit flexors (Goldmann et al., 2013; Kurihara et al., 2014; Yamauchi and Koyama, 2019). Whilst these approaches provide a broad understanding of toe flexion strength, they may not accurately represent the differential actions of different muscle groups that underpin the foot's function. Failing to acknowledge the muscle groups independently, and in turn the different orientation of the MTP joint axes about which the toes rotate (Bojsen-Møller and Lamoreux, 1979), may misrepresent the joints' mechanical functions (Smith et al., 2012) and role of the muscles involved. This is reinforced by the findings of Saeki et al. (2021), who showed that maximal voluntary contraction (MVC) torque about the first MTP joint was affected differently by ankle position to MVC torque about the lesser digits. This suggests an interaction between force contributions of intrinsic versus extrinsic muscles and the MTP joints upon which they act. Furthermore, comparative work has shown that, while the several toe flexors of humans are similarly or relatively larger sized than their equivalents in great apes (Oishi et al., 2018; Vereecke et al., 2005), the extrinsic digital flexor (flexor fibularis) of chimpanzees and bonobos is considerably larger than the human flexor digitorum longus (Holowka and O'Neill, 2013; Vereecke et al., 2005) (Table S1 and Fig. S1). This suggests some differential functional adaptation in humans and a complete understanding of human toe flexor strength requires a breakdown of intrinsic, extrinsic, hallucal and digital flexors. While Saeki et al.’s (2021) work moved in this direction, the authors employed voluntary contractions that may not accurately capture the full force-generating potential of foot muscles (Olivera et al., 2020). They also did not fully parameterise the active and passive torque–angle relationships across the full range of joint motion. Using supramaximal electrical stimulation techniques to maximally activate muscles and parameterising the wider passive and active torque–angle relationships could better capture foot muscle capabilities. This would enhance our understanding of foot muscle contributions to human locomotion and enable more informed musculoskeletal modelling of human feet to mechanistically link foot muscle contraction to walking or running performance.

Therefore, our first aim was to compare the maximal electrically evoked MTP joint torque produced by plantar intrinsic muscles with that of all plantar foot muscles, across the range of motion of the MTP joint, and parameterise the torque–angle relationships. We hypothesised that optimal MTP joint angles for torque production would reflect those angles where MTP joint torque is greatest during gait. Our second aim was to compare the electrically evoked active muscle torques with passive torque contributions (including passive muscular and ligamentous contributions). The latter was to capture the potential for active muscular contractions to contribute alongside passive force development within the foot during gait. We hypothesised that, at MTP joint angles commonly used in gait, maximal active muscle torques would be larger than passive torques, reflecting the functional importance of active muscular contraction within the foot.

To achieve our aims, we determined toe-flexion torque–angle relationships for the PIMs, extrinsic foot muscles and passive structures acting on the hallux and lesser toes. This involved development of a custom experimental setup, coupled with motion capture and a supramaximal stimulation protocol to isolate PIMs torque from extrinsic muscle torque for both the hallux and lesser digits, and evaluation of the reliability and repeatability of this approach for experimental rigour.

## MATERIALS AND METHODS

### Participants

Nine healthy participants (6 men, 3 women; mean±s.d. age 28±5 years, height 174±7 cm, mass 71±11 kg) with no history of diagnosed lower limb or foot injuries in the 6 months prior to data collection provided written informed consent to participate in the study that was approved by the University of Exeter ethics committee. The required sample size for a repeated measures analysis of variance (ANOVA) was informed by the effect of ankle angle and MTP joint angle on MTP joint torque reported by Saeki et al. (2021) [partial eta squared=0.31; effect size (Cohen's *f*)=0.67; α error=0.05; power (1−β)=0.85; correlation among repeated measures=0.5; *n*=8] and calculated using G*Power 3.1 (Heinrich Hein University, Dusseldorf, Germany) to give an *n* of 8.

#### Experimental setup

**Fig. 1.**
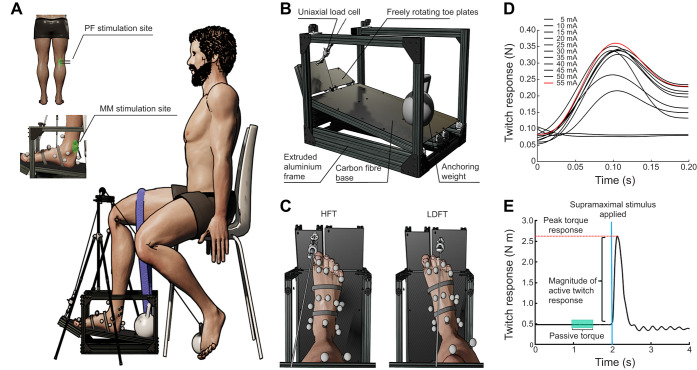
**Schematic representation of the experimental setup.** (A) Position of the participant in the rig setup, seated upright with the knee flexed and the ankle in a neutral position. The angle of the MTP joint is manipulated via rotation of the extended toe-plate. The arrangement of the bipolar electrodes at both the popliteal fossa (PF) and medial malleolus (MM) stimulation sites is displayed. (B) Custom-built foot rig instrumented with a uniaxial force transducer that attaches to carbon-fibre plates designed to measure hallux torque and lesser digit torque independently. (C) Foot placement for the hallux flexion torque (HFT) and lesser digit flexion torque (LDFT) measurements; the axes of the metatarsophalangeal (MTP) joints align with the rotational axes of the respective toe-plates. Once positioned, the heel and toes were marked with tape to keep foot position consistent within conditions. The foot's position within the rig was secured using tensioned adjustable straps across the midfoot, forefoot and the required digit(s) that were fed through small slots in the foot plate to be tightened beneath. Further heavy-duty taping was employed over the strapping and alongside the foot to ensure secure and stable positioning of the foot for HFT and LDFT measurements. (D) Representative participant data to demonstrate the process to identify the current required to elicit supramaximal stimulation for each experimental condition. Each curve represents the twitch response to a single electrical pulse. Current was incrementally increased by 5 mA until the twitch response plateaued, in this case at 55 mA (red). Subsequent stimulations applied during the experimental protocol were amplified by a further 20% to ensure twitches were supramaximal. (E) Representative raw data highlighting an individual twitch contraction. Passive torque measurement was determined by averaging the baseline signal (teal shading) prior to stimulus delivery (blue line). Peak active torque was then determined by subtracting the passive torque measure from the peak total torque of each stimulated twitch response (red dashed line).

Separate isometric hallux (HFT) and lesser digit (LDFT) flexion torque–angle data were collected using a custom-built foot rig (Fig. 1B) on two occasions. The rig, featuring two freely rotating, carbon-fibre plates extending from its frame, was specifically engineered to quantify hallux torque data and lesser digit torque data independently. Participants sat upright in a supported chair, with the knee flexed and the ankle in a neutral position (Fig. 1A). The participant's right foot was positioned within the rig according to the MTP joint condition. For HFT measurements, the transverse axis of the first MTP joint was parallel to the rotational axis of the first extended toe plate (Fig. 1C). During LDFT measurements, the foot was repositioned such that the oblique axis of the lesser-digit MTP joints (Bojsen-Møller and Lamoreux, 1979) was parallel to the rotational axis of the second toe-plate (Fig. 1C). Within each condition, the foot was marked at the heel and toe to ensure foot placement was consistent throughout the procedure. Once positioned appropriately within the rig, the foot was secured with strapping across the forefoot, midfoot, rearfoot and ankle joint to limit unwanted movement artefact during contraction to minimise the change in ankle and MTP joint moment arms. The rig was anchored to the ground so that it could not slip during experimental trials. The left foot was placed on the ground beside the rig set-up. Each participant was familiarised with the setup prior to testing as the toes were passively rotated through their angular range of motion.

The rig was instrumented with an inline uniaxial force transducer with a 0–100 N range and <0.03 N accuracy (DDE-100N-002-000, Applied Measurements Ltd, Reading, Berkshire, UK) (Fig. 1B) that was connected to the toe plate via a steel cord. The cord passed over a pulley system and was anchored behind the participant. The angle of the toe plate was adjusted by moving the anchor forwards or backwards. External joint flexion torque was then calculated as the product of the recorded force magnitude from the transducer multiplied by the external moment arm (Eqn 1):
(1)

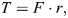
where *T* is the external joint flexion torque assumed to represent the combined contribution of both active and passive structures active about the MTP joint(s) at the measured angle, not the intended angle; *F* is the force measured by the uniaxial load cell; and *r* represents the external moment arm defined by the perpendicular distance from the MTP joint centre to the force vector. The MTP joint centre was determined as the location of the joint centre within the kinematic model (see ‘Kinematic and kinetic analysis’, below), transformed into the global coordinate system.

#### Experimental protocol

Within both HFT and LDFT conditions, isometric toe flexion was elicited via supramaximal transcutaneous electrical stimuli applied to the tibial nerve (see ‘Peripheral nerve stimulation’, below). To distinguish PIM-generated flexion torque contributions from the extrinsic toe flexors, the stimulus was delivered at two locations of the tibial nerve: the popliteal fossa (PF) and posterior to the medial malleolus (MM) as depicted in Fig. 1A. The former was to elicit contraction of all muscles innervated by the tibial nerve (PIMs, gastrocnemius, soleus, plantaris, popliteus, tibialis posterior, flexor hallucis longus and flexor digitorum longus) and the latter only the PIMs. Thus, four specific contraction conditions were established to describe specific toe-flexion torque–angle curves: HFT_PF_, HFT_MM_, LDFT_PF_ and LDFT_MM_. Within each condition, the angular orientation of the toe plate was adjusted to manipulate the pose of the respective MTP joints through each participant's range of motion. The external torque was recorded from multiple MTP joint angles across the range of motion; typically, 8–12 joint angles in ∼5 deg increments, totalling approximately 40 contractions throughout the procedure. Participants received approximately 60 s of rest between each contraction to minimise the effects of fatigue. The order of experimental conditions was pseudo-randomised whilst the order of measurement angles was randomised until the range of motion had been moved through twice. Each participant completed the procedure on two separate occasions to determine the reliability and repeatability of the experimental setup.

#### Peripheral nerve stimulation

We adapted a previous peripheral tibial nerve stimulation protocol (Hoffman et al., 2012, 2014; Pincheira et al., 2018) to examine the torque–angle relationship for each contraction condition. At the beginning of each data collection session, with the participant's foot positioned with the toes at a neutral angle within the rig, the amount of electrical current required to achieve supramaximal stimulation of the tibial nerve was determined from both the PF and MM sites. This procedure was repeated for both HFT and LDFT foot positions. A single pulse constant current stimulus (500 µs, DS75-55, Digitimer, Welwyn Garden City, UK) was applied transcutaneously to the tibial nerve via a pair of surface electrodes (Ag–AgCl electrode, FS-TC1/6, 35 mm diameter, Skintact) positioned at the stimulation site. Current was passed from a cathode placed on the stimulation site to a proximal anode. Optimal electrode location was carefully determined by palpating the tibial nerve at the posterior knee and delivering a series of small single electrical pulses via a Digitimer Compex Motor Point Pen electrode (4 mm diameter, Digitimer). The participant's physical response alongside the force applied to the load cell was monitored to confirm that the stimulus elicited toe flexion. Once the optimal site was identified and the bipolar electrodes were attached to the participant, the intensity of the stimulus required to elicit a maximal response was identified. During this process, the intensity of the current was gradually increased from a minimal level until the magnitude of resting twitch force reached a plateau (measured by the load cell) (Fig. 1D). The maximal current intensity for each experimental condition was determined using this protocol, and then further increased by 20%. This procedure was repeated separately for HFT and LDFT configurations. To determine torque–angle relationships for each condition, a triplet of supramaximal pulses (50 Hz) was applied to evoke resting torque twitches at each MTP joint pose throughout the range of motion.

### Data acquisition

Isometric toe flexion force was recorded by the uniaxial load cell at 2000 Hz using a 16-bit analog-to-digital converter (ADC USB-2533, Qualisys, Gothenburg, Sweden), and Qualisys Track Management software. Three-dimensional motion capture data were synchronously collected at 200 Hz using an 8-camera opto-electronic system (MIQUS, Qualisys). Twenty-three retroreflective markers (8 mm diameter) were attached over anatomical landmarks of the right knee, shank and foot of participants in accordance with the Istituto Ortopedici Rizzoli (IOR) multi-segment foot model (Leardini et al., 2007). Two additional markers were affixed to the most proximal and distal ends of the uniaxial force transducer to define the orientation of the exerted force.

### Data analysis

#### Kinematic and kinetic analysis

Resultant twitch force data were digitally filtered using a 20 Hz recursive second-order low-pass Butterworth filter in MATLAB (MATLAB2021b, The MathWorks Inc., Natick, MA, USA). Positional data from the two retroreflective markers on the load cell shaft were used to determine the orientation of the load cell and compute the force vector and the magnitude of the resultant external torque. Anatomical marker trajectories were exported to OpenSim v.4.2 software (Seth et al., 2018) to define and scale a joint-constrained rigid body multi-segment model of the shank, calcaneus, midfoot, metatarsal and toes for each participant (Maharaj et al., 2022). This model shows good agreement with biplanar videoradiography measurements of foot kinematics (Maharaj et al., 2022). We adapted the model such that the existing single ‘toes segment’ was split into two separate segments; the hallux, operated on a transverse axis aligned through the first and second metatarsal head, and the lesser toes, operated on an oblique axis defined from the second and fifth metatarsal heads (Bojsen-Møller and Lamoreux, 1979). Markers placed on the proximal phalanx of the hallux and on the fourth digit were used to track the motion of the hallux and lesser toe segments, respectively. The orientation of the hallux and lesser toe segments relative to the lumped metatarsal segment was used to calculate hallux angle and lesser toe angle. OpenSim inverse kinematics analysis (Delp et al., 2007) was implemented to determine foot kinematics during trials and observe any rearfoot motion induced by twitch contractions. Specifically, medial longitudinal arch angle, defined as the vector angle between anatomical markers situated on the medial calcaneus, navicular and first metatarsal head, and ankle angle, defined as the angle of the talus relative to the tibia within the musculoskeletal model (Maharaj et al., 2022) were quantified to determine whether any angular displacement occurred as a result of the electrical stimulation. Inverse kinematic data were then filtered using a 10 Hz recursive second-order low-pass Butterworth filter. The perpendicular distance from the MTP joint centre to the force vector was computed to determine the external moment arm (*r*). The joint centre location was determined as the three-dimensional position of the kinematic model's MTP joint, transformed into the global coordinate system. External joint flexion torque was then calculated for each trial as described previously (Eqn 1).

#### Torque–angle curve construction

**Fig. 2.**
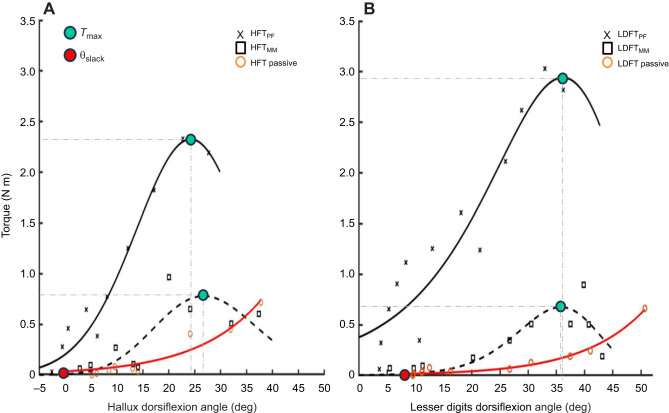
**Representative individual torque–angle relationships from a single lab visit.** Data points and fitted active (solid black line, popliteal fossa, PF; dashed black line, medial malleolus, MM) and passive (solid red) torque–angle curves from isometric hallux (A) and lesser digit (B) flexion presented from a representative single visit (*n*=1). Maximum torque (*T*_max_) and optimal joint angle (θ_opt_) are identified from the vertical *y*-axis and horizontal *x*-axis values, respectively, at each active curve's apex. For the passive curve, the slack angle (θ_slack_) is indicated by a red circle.

Active and passive torque–angle curves were constructed for each condition using methods described previously (Hoffman et al., 2012, 2014; Pincheira et al., 2018). The modelled MTP joint angle corresponding to peak twitch torque was plotted against the peak active torque derived from the triplet stimulation. Passive torque–angle curves were first computed from the average resting joint torque recorded during the first 1.0–1.5 s of each trial, prior to the delivery of the stimulus (Fig. 1E). A third-order polynomial function was then fitted to the passive torque–angle data (Fig. S1A). The function was then evaluated to identify the angle corresponding to 0 N m of torque – hereafter referred to as the slack angle (θ_slack_). An exponential function (Eqn 2) was then fitted to all passive torque–angle data beyond θ_slack_ (Fig. 2; Fig. S2), with *T*_passive_ representing passive torque, θ denoting the modelled MTP joint angle and exponential coefficients, *k* indicating the stiffness of the curve representing passive stiffness of the MTP joint(s), and *A* indicating its curvature. It should be noted that these passive data include contributions from all passive structures, particularly the PA, and therefore the data are not reflective of the passive force from the muscles involved:
(2)

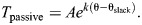
Active torque was subsequently computed as the difference between peak torque of the twitch response and the passive torque determined by evaluating the above function at the corresponding MTP joint angle (Hoffman et al., 2012) (Fig. 1E). An active torque–angle function (Eqn 3) was fitted to the active torque–angle data using a physiologically appropriate model (Azizi and Roberts, 2010; Otten, 1987):
(3)

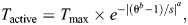
where *a*, *b* and *s* represent the roundness, skewness and width of the curve, respectively. Maximum torque (*T*_max_) and the MTP joint angle corresponding to *T*_max_ (optimal MTP angle, θ_opt_) were determined from the fitted torque–angle curves (Fig. 2). Average active and passive curve parameters were computed from each participant, and mean torque–angle curves were produced using the averaged coefficients to determine the curve fit for each experimental condition.

### Statistical analysis

From the fitted torque–angle functions, dependent variables (*T*_max_, θ_opt_, *k*, θ_slack_) from each visit were determined for each participant (see Dataset 1). Data normality was assessed using the Shapiro–Wilk test and subsequently analysed using a two-way repeated measures ANOVA within a statistical software package (SPSS statistics 28, IBM, Chicago, IL, USA). Visit (V1 and V2) and stimulation site (PF and MM) were defined as factors within the ANOVA. An alpha level of 0.05 was set for statistical significance for all tests. *A priori* planned comparisons (Bonferroni contrasts) were incorporated into the ANOVA design to compare the effects of stimulation site and MTP joint over the variables in the analysis.

To evaluate the test–retest reliability, the intraclass correlation coefficient (ICC; 2,*k*; absolute agreement), standard error of measurement (SEM) and minimum detectable change MDC_95%_ were calculated from torque–angle parameters estimated between visits using custom MATLAB scripts. The ICC (2,*k*; absolute agreement) model was estimated for repeated torque–angle parameter measurements (2 measurements) based on a two-way mixed-effects model to determine the absolute agreement between repeated measures. ICC cut-off values outlined by Koo and Li (2016) were used to interpret the ICC as a measure of reliability (i.e. <0.5=‘poor’; 0.50–0.75=‘moderate’; 0.75–0.90=‘good’; and >0.90=‘excellent’). The SEM for each parameter was calculated as SEM=s.d.×√(1−ICC), where s.d. is the standard deviation of the measurements. The MDC_95%_ was calculated as MDC_95%_=SEM×1.96×√2, where 1.96 is the *z* score for a 95% confidence interval (Haley and Fragala-Pinkham, 2006) and provides a threshold value for determining whether observed changes in torque–angle parameters reflect true differences rather than random measurement error or variability. The MDC_95%_ values reported here establish what constitutes a measurable difference in future experiments using such a setup for intervention or comparison studies. Additionally, to indicate the proportion of variation between the two visits, the ratio of the difference between visits to the average of the two visits is presented for each torque–angle parameter.

## RESULTS

### Hallux flexion torque–angle relationships

Torque–angle relationships of hallux flexors exhibited an ascending–descending profile under both supramaximal stimulation conditions (Fig. 2). PIM-only *T*_max_ ranged from 0.57 to 1.76 N m, with θ_opt_ ranging between 23.4 and 67.3 deg. When extrinsic muscle contributions (HFT_PF_) were included, *T*_max_ values ranged from 2.03 to 4.02 N m, with θ_opt_ occurring between 24.2 and 70.6 deg. Similarly, high inter-individual variability was recorded with passive curves in θ_slack_ (3.3–35.5 deg) and *k* (0.05–0.17). Mean torque–angle parameters from each respective visit are expressed in Table 1.

**Table 1.** Summary of repeated measures mean torque–angle curve parameters and inter-day reliability analysis for each stimulation condition and subsequently calculated torque–angle curve coefficients

	Visit 1	Visit 2	Ratio of difference from mean	ICC	SEM	MDC_95%_
HFT_PF_						
*T*_max_ (N m)	3.04±0.68	3.06±0.76	0.13±0.12	0.83	0.29	0.79
θ_opt_ (deg)	48.24±14.63	47.69±13.29	0.21±0.12	0.84	5.37	14.9
*k*	0.1±0.04	0.09±0.02	0.25±0.19	0.67	0.02	0.05
θ_slack_ (deg)	22.64±8.86	23.04±9.66	0.15±0.15	0.97	1.52	4.22
HFT_MM_						
*T*_max_ (N m)	1.04±0.40	0.94±0.31	0.42±0.22	−0.24	0.39	1.09
θ_opt_ (deg)	47.39±13.16	45.55±14.44	0.13±0.12	0.93	3.45	9.57
*k*	0.1±0.04	0.09±0.02	0.25±0.19	0.67	0.02	0.05
θ_slack_ (deg)	22.64±8.86	23.04±9.66	0.15±0.15	0.97	1.52	4.22
LDFT_PF_						
*T*_max_ (N m)	3.17±1.05	3.22±0.97	0.22±0.17	0.81	0.42	1.18
θ_opt_ (deg)	42.42±13.66	42.83±12.49	0.14±0.09	0.93	3.28	9.09
*k*	0.09±0.02	0.08±0.02	0.21±0.20	0.68	0.01	0.04
θ_slack_ (deg)	16.21±11.39	18.25±11.14	0.19±0.26	0.98	1.54	4.28
LDFT_MM_						
*T*_max_ (N m)	1.34±0.36	1.22±0.35	0.10±0.10	0.94	0.09	0.25
θ_opt_ (deg)	39.56±13.20	39.92±12.98	0.18±0.11	0.88	4.33	12.01
*k*	0.09±0.02	0.08±0.02	0.21±0.20	0.68	0.01	0.04
θ_slack_ (deg)	16.21±11.39	18.25±11.14	0.19±0.26	0.98	1.54	4.28

Data are means±s.d. (*n*=9). ICC, intraclass correlation coefficient; SEM, standard error of measurement; MDC_95%_, minimum detectable change (95% confidence interval); HFT, hallux flexion torque; LDFT, lesser digit flexion torque; PF, popliteal fossa; MM, medial malleolus. Units of SEM and MDC_95%_ are consistent with measurement units (*T*_max_: N m; angular parameters θ_opt_ and θ_slack_: deg).

**Fig. 3.**
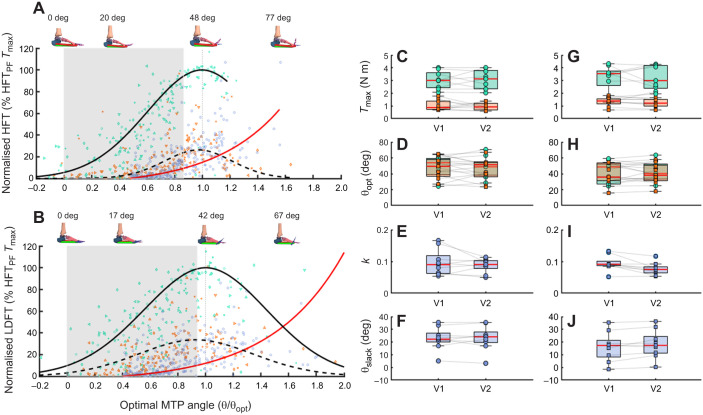
**Group mean toe-flexion torque–angle relationships.** Active (solid black line, PF site stimulation; dashed black line, MM site stimulation) and passive (red) torque–angle curves of hallux flexors (A) and lesser digit flexors (B) fitted through normalised individual data points (teal, PF site; orange, MM site) (*n*=9). Each MTP joint angle was normalised to the calculated optimal joint angle (θ_opt_), and torque was normalised to the calculated maximum stimulated twitch torque produced by a triplet of stimuli at 50 Hz (*T*_max_) within each PF condition. Passive torque–angle data were also normalised to the same parameters, with the curve originating from the mean calculated slack angle (θ_slack_) for each respective joint. Grey shading indicates typical MTP joint angle range during gait (Falisse et al., 2022), normalised to θ_opt_ of HFT_PF_ (A) and LDFT_PF_ (B), respectively. Active (PF, teal; MM, orange) and passive (blue) hallux flexor (C–F) and lesser digit (G–J) torque–angle curve parameters are presented as boxplots from each lab visit (V1, visit 1; V2, visit 2) showing the median of individual data points and the upper and lower interquartile range, with individual parameter values plotted indicating variability between participants. *T*_max_, maximum torque; θ_opt_, optimal MTP angle; *k*, passive torque–angle relationship stiffness coefficient; θ_slack_, MTP angle corresponding to 0 N m of passive torque.

Participants produced significantly greater *T*_max_ when the extrinsic muscles were innervated alongside the PIMs (group mean±s.d.: HFT_PF_ *T*_max_=3.05±0.70 N m, HFT_MM_ *T*_max_=0.99±0.35 N m, *F*_1,8_=76.7, *P*<0.01) with a mean increase of 2.06 N m (95% confidence interval, CI, 1.52–2.60 N m) when the extrinsic toe flexors were engaged (Fig. 3A,C). Despite the increase in *T*_max_, stimulation site had no effect on θ_opt_ (*F*_1,8_=0.61, *P*=0.46) (Fig. 3D). Passive torque contributions were initiated with 22.6±8.9 deg MTP joint dorsiflexion with comparable passive curve parameters *k*, and θ_slack_ between conditions and visits (Fig. 3E,F).

### Lesser digit flexion torque–angle relationships

Torque–angle relationships of lesser digit flexors mirrored those of the hallux, displaying an ascending-descending profile in both supramaximal stimulation conditions (Fig. 2). *T*_max_ ranged from 0.67 to 1.83 N m with θ_opt_ ranging from 16.0 to 58.1 deg when only stimulating PIMs and from 1.28 to 4.33 N m, with θ_opt_ occurring between 24.5 and 63.5 deg when the extrinsic toe flexors were also stimulated. Similarly, high inter-individual variability was recorded with passive curves in θ_slack_ (−1.3–36.4 deg) and *k* (0.05–0.13). Stimulating from the PF produced stronger lesser digit contraction compared with MM stimulations (group mean±s.d.: LDFT_PF_ *T*_max_=3.20±0.98 N m, LDFT_MM_ *T*_max_=1.28±0.35 N m, *F*_1,8_=44.3, *P*<0.001; Fig. 3B,G) with a mean increase of 1.92 N m (95% CI 1.25–2.58 N m) when the extrinsic toe flexors were also engaged. However, stimulation site had no significant effect on mean θ_opt_ (*F*_1,8_=0.61, *P*=0.46; Fig. 3H). Lesser digit θ_slack_ showed passive torques generated from 16.2±11.39 deg MTP dorsiflexion with similar passive curve parameters *k*, and θ_slack_ between conditions and visits (Fig. 3I,J).

### Reliability of torque–angle curve construction methods

The test–retest reliability of torque–angle curves constructed from the employed methods, repeated measures of active curve parameters *T*_max_ and θ_opt_, and passive curve parameters *k* and θ_slack_ were evaluated within each condition. Reliability statistics of all torque–angle relationships and their coefficients are presented in Table 1. Notably, 11 of 16 torque–angle parameter measurements exhibited good to excellent test–retest reliability [ICC(2,*k*)=0.75–0.98], whilst 4 out of 16 displayed moderate to good test–retest reliability [ICC(2,*k*)=0.5–0.75] aligning with the standards established by Koo and Li (2016).

Apart from the HFT_MM_ condition, *T*_max_ demonstrated good to excellent test–retest reliability across all conditions. Within the HFT_MM_ condition there was greater proportion of variability between visits compared with the mean of both visits (Table 1), contributing to the poor reliability measure. This can likely be attributed to a greater between-mean squares disparity than within-mean squares, despite the small SEM (0.39 N m) and MDC_95%_ values (1.09 N m). For optimal MTP joint angle (θ_opt_), *k* and reliability measurements were consistent across stimulation conditions. Low SEM values relative to the mean torque–angle parameters (*T*_max_­ SEM≈17%, θ_opt_­ SEM≈9%, *k*­ SEM≈16%, θ_slack_­ SEM≈8%), and low MDC_95%_ indicate good precision in the measurements, despite high inter-individual variation.

The kinematic analysis highlights that the strapping applied to the foot and ankle structures successfully restricted foot and ankle movements throughout the experimental protocol (Table 2). This restriction resulted in minimal angular displacement of the medial longitudinal arch irrespective of stimulation site. Similarly, the strapping also prevented substantial plantarflexion of the ankle joint.

**Table 2.** Group mean (±s.d.) longitudinal arch and ankle joint angular range of motion during supramaximal twitch contractions in each condition

	Longitudinal arch compression (deg)	Ankle plantarflexion (deg)
HFT_PF_	0.7±0.5	3.6±1.2
LDFT_PF_	0.5±0.3	4.2±1.4
HFT_MM_	0.7±0.5	0.3±0.1
LDFT_MM_	0.4±0.3	0.6±0.2

## DISCUSSION

The mechanical requirement to produce torque about the MTP joints has been considered important for human bipedal gait (Bojsen-Møller and Lamoreux, 1979; Bramble and Lieberman, 2004; Holowka et al., 2021; Pontzer, 2017; Susman, 1983). The necessary force production has been attributed to both active and passive structures of our feet during tasks such as walking and running (Farris et al., 2019; Kelly et al., 2019; Riddick et al., 2019; Smith et al., 2022a). However, our understanding of the mechanisms governing this ability, and the relative contributions of active and passive structures is incomplete. Here, we have quantified the torque–angle relationships of anatomically distinct muscle groups acting on the MTP joints and separated active and passive joint torque contributions. We showed that at MTP joint angles relevant to gait, maximal torques generated by plantar extrinsic and intrinsic muscles were considerably larger than those from passive tissues (including passive muscle force), with extrinsic muscles offering the greatest torque potential. We also showed that the MTP joint angles corresponding to the ascending limb of the torque angle relationship are the same range through which the MTP joint operates in gait. The former finding suggests a prominent role for plantar foot muscles in MTP joint torque production, and the latter indicates that these same muscles are well positioned to apply those torques to stiffen the distal foot during gait.

To achieve the above, we employed a novel, *in vivo* technique to describe the torque-producing ability of the foot's plantar musculature. To provide confidence in this method, we quantified the inter-day reliability of torque–angle curve parameters. Using a combination of supramaximal nerve stimulation and a purpose-built foot rig, we have shown reliable and repeatable torque–angle curves and coefficients of both PIM-only and PIM plus extrinsic hallux and lesser digit flexion with moderate to excellent inter-day reliability (Table 1, Fig. 3) (Koo and Li, 2016). Although the ratio of the mean differences between visits to mean values was quite high (15–25%), this is largely due to the relatively low absolute torque values and equates to quite small absolute differences (0.4–0.75 N m). While others have partially quantified the active MTP joint torque–angle relationship in a similar setup via maximal voluntary contractions (Saeki et al., 2021), our use of transcutaneous nerve stimulation and application of physiological models to the data allowed us to more confidently parameterise the wider torque–angle relationship and compare among functional groups of torque-generating tissues.

### Torque–angle parameters mapped to walking and running kinematics

Torque–angle relationships broadly identify the operating region of a muscle or group of synergistic muscles acting about a joint on the force–length relationship (Hahn et al., 2011; Maganaris, 2001). Typically, the MTP joints of healthy individuals dorsiflex approximately 40–50 deg beyond neutral as we transition through midstance to the propulsive phase of the gait cycle (Bojsen-Møller and Lamoreux, 1979; Farris et al., 2020; Welte et al., 2021), with peak joint moments occurring immediately prior to toe-off to stiffen the forefoot for effective push-off (Farris et al., 2019, 2020). The active torque–angle curves from the current study show the operating region of the PIM and extrinsic toe flexor muscle groups during gait correspond to angles where active force production is favoured. We state this because each curve's ascending limb corresponds to angles at which there is an increasing need for joint torque during gait (Fig. 3A,B). Peak active hallux and lesser digit isometric torques were achieved at slightly more dorsiflexed MTP joint angles (hallux: 48.0±13.6 deg; lesser digits: 42.6±13.4 deg) than the angles at which peak MTP joint moments are observed in gait. This suggests that these muscles act on their ascending limb during gait and are close to the top of the ascending limb when the greatest MTP joint torque is required for stiffening the MTP joint during push-off. While we cannot state conclusively that plantar foot muscles of humans have evolved specifically for this function, the findings do suggest that their structure is well adapted to contribute to it. Although comparative data are scarce, Vereecke et al. (2005) dissected gibbon and bonobo foot muscles, noting that the human flexor hallucis brevis is considerably larger, and the transverse head of the adductor hallucis is relatively smaller. Vereecke et al.’s (2005) interpretation was that these evolved differences reflect the greater importance of toe flexion strength for human bipedal walking. Their conclusions are somewhat supported by Oishi et al. (2018), who found that human plantar intrinsic muscles were similarly large or, in the case of the quadratus plantae, larger than those of other great apes. Comparisons of extrinsic muscles are slightly hampered by the different anatomy in humans and other apes, whereby the human flexor hallucis longus and flexor digitorum longus muscles act separately on the hallux and lesser digits, whereas the flexor tibialis and flexor fibularis muscles identified in chimpanzees, bonobos and gibbons attach to phalanges 1 and 5 (flexor tibialis) and 2–4 (flexor fibularis), respectively. The flexor hallucis longus and flexor tibialis both attach from the tibia to the hallux and are of comparable size in humans and other apes (as summarised in Table S1 and Fig. S2), whereas flexor fibularis data from chimpanzees (Holowka and O'Neill, 2013) and bonobos (Vereecke et al., 2005) suggest this muscle has a much higher physiological cross-sectional area than the flexor digitorum longus in humans. Together, this could mean that human evolution has preserved flexion strength of the first MTP joint and not the other MTP joints. Our data do not support this because similar maximal torques were observed for HFT and LDFT (Table 1). However, functional strength for a specific task depends on not just muscle size but also where on a muscle's force–length or torque–angle relationship it operates during that task. Our data support the notion that human toe–flexor muscle structure is well adapted to the need for generating toe flexion torque in the propulsive phase of gait by highlighting that these muscles operate on a favourable region of their torque–angle curve. While the torque at the MTP joint may appear modest compared with those generated for propulsion elsewhere in the lower limb, it has been previously demonstrated in human walking, running and jumping that removing the active muscular portion of this torque via tibial nerve blocks perturbs lower limb joint power production (Farris et al., 2019; Smith et al., 2022b).

It should be noted that we only manipulated MTP joint angles, and the length of plantar foot muscles is also influenced by foot arch compression and recoil that occurs during the stance phase of gait (Kelly et al., 2014, 2015). This influence challenges the direct mapping of the torque–angle relationship established here to MTP joint angles during gait and is an inherent limitation of interpreting torque–angle relationships for multi-articular muscles. However, during the stance phase of gait, the foot's longitudinal arch is generally under load, causing the arch to compress and elongate (Kelly et al., 2015). Consequently, the plantar foot muscles would be slightly longer for any given MTP joint angle when compared with our data, which were collected with an unloaded foot. This would have the effect of shifting the torque–angle curve from Fig. 3A,B leftward in relation to the range of angles from gait (grey shading in Fig. 3A,B). As a result, the muscles would be operating more on the top of the ascending limb and optimal plateau region, and therefore this would not change our conclusions.

The extrinsic muscles' force–length relationships would also be affected by ankle joint angle, which was fixed in our testing but would vary through the stance phase of gait. Plantar flexion of the ankle during push-off therefore might cause extrinsic toe flexors to operate at shorter lengths than our data would predict. This would not greatly influence any passive force contributions but could move active contraction of extrinsic muscles to a less-favourable portion of their force–length relationship. To map the torque–angle relationships for different ankle joint angles, all current twitch contractions would need to be repeated for a range of ankle joint positions and would have required too many electrical stimulations. Therefore, we opted to fix a neutral ankle angle and focus on the MTP joint, which influences all the muscles and tissues being investigated. However, extension of the current work to a greater range of ankle positions would be valuable in future.

### Comparing active and passive contributions to MTP joint torque

The magnitude of external MTP joint torque was dominated by the foot's muscles at angles relevant to the propulsive phase of gait (0–40 deg dorsiflexion; Falisse et al., 2022) across all supramaximal contraction conditions (Fig. 3). In part, the strength of muscle contraction depends on the volume of skeletal muscle innervated and the number of motor units recruited to generate the muscular response (Fukunaga et al., 2001). Therefore, it is no great surprise that *T*_max_ was significantly greater from PF-stimulated contractions (HFT_PF_ *T*_max_=3.05±0.70 N m, LDFT_PF_ *T*_max_=3.19±0.98 N m) than from MM-stimulated contractions (HFT_MM_ *T*_max_=0.99±0.35 N m, LDFT_MM_ *T*_max_=1.28±0.34 N m), considering the combined muscle volume of PIM hallux flexors (abductor hallucis, flexor hallucis brevis, 48.46±8.69 cm^3^), lesser digit toe flexors (flexor digitorum brevis, quadratus plantae, abductor digiti minimi 44.17±8.69 cm^3^) and the additional activation of the extrinsic toe flexors (flexor hallucis longus, flexor digitorum longus 88.60±13.74 cm^3^) determined from 1.5 T MR imaging (Kusagawa et al., 2022). Equally, comparable *T*_max_ values observed when stimulating only intrinsic hallux (HFT_MM_) and lesser digit flexors (LDFT_MM_) may be attributed to the comparable muscle volumes acting about each MTP joint (Franettovich Smith et al., 2021; Kusagawa et al., 2022). *T*_max_ values were also similar for hallux and lesser digits within the respective PF conditions, suggesting similar maximal extrinsic muscle torque capacity for both. This is despite reports that the extrinsic muscles acting on the hallux are larger than those acting on the lesser digits (Kusagawa et al., 2022). However, there is disparity over extrinsic foot muscle volume ratios (Green and Briggs, 2013; Ichikawa et al., 2021; Kusagawa et al., 2022) and it is possible our group had more similar muscle volumes between extrinsic hallux and lesser digit flexors as shown in Green and Brigg's (2013) study as opposed to Kusagawa et al.’s (2022) study. It may also be possible that similar torque outputs are achieved despite a morphological disparity between the extrinsic hallux and lesser digit flexors as a result of the moment arms of the individual muscles influencing the torque output.

Notably, the magnitude of *T*_max_ determined within each experimental condition of our study aligns closely with maximal MTP joint flexion moments theoretically determined by Green and Briggs (2013). Their methods involved MR imaging of the foot's muscles to determine cross-sectional area before quantifying maximal muscle force using widely accepted reference values of specific tension (25–40 N cm^−2^), and subsequently estimating flexion moment as the product of maximal muscle force and moment arm length measured from MRI data. Specifically, the flexion moment of PIMs operating about the first MTP joint (1.05–1.68 N m) calculated by Green and Briggs (2013) is similar to our measured value for HFT_MM_ *T*_max_ (0.99±0.35 N m). Furthermore, by including the flexor hallucis longus, the first MTP joint flexion moment increased (2.88–4.61 N m; Green and Briggs, 2013), aligning well with our value for HFT_PF_ *T*_max_ (3.05±0.70 N m). Similarly, our LDFT_MM_ *T*_max_ values (1.28±0.34 N m) closely match the combined flexion moment of PIMs acting on the second to fifth MTP joints (1.10–1.76 N m), as per Green and Briggs (2013), and with the inclusion of the flexor digitorum longus (2.29–3.67 N m), flexion moments matched with LDFT_PF_ *T*_max_ measurements (3.19±0.98 N m) within the current study. This congruence lends additional credibility to the torque measurements and interpretation of the results in both studies.

Furthermore, our results are consistent with previous work reporting a significant reduction in external MTP joint moment when the force-producing capacity of the extrinsic toe flexors was inhibited (Goldmann and Brüggemann, 2012). Despite only achieving ∼35% of PF-stimulated *T*_max_, PIM-only torque–angle curves highlight that active muscular torque remained the dominant net contributor until MTP joint dorsiflexion increased to 1.1 θ_opt_ (Fig. 3A,B). This shows that even the force-generating potential of the PIMs alone is greater than that of passive tissues during tasks such as walking, adding to the growing evidence that active muscular contributions rather than passive mechanisms augment the foot's mechanical ability (Farris et al., 2019, 2020; Kelly et al., 2019; Riddick et al., 2019, 2021; Smith et al., 2022b). However, it must be noted that we supramaximally electrically stimulated the muscles, and contributions during gait will depend on sub-maximal activation levels.

The relatively low passive torques observed within our data (assumed to be produced from strain placed upon the PA, other ligaments and passive muscle stretch) throughout the range of hallux and lesser digit MTP joint motion may offer insight into the passive function of the foot. That passive torques only became dominant once MTP joints were dorsiflexed beyond 1.1–1.5 times θ_opt_ (equating to 52–73 deg hallux and 45–62 deg lesser digit dorsiflexion, respectively) adds to debate over the strength of the association between the windlass mechanism [passive tensioning of the midfoot via toe dorsiflexion about the MTP joints, which exerts a pull on the calcaneus through the PA, as purported by Hicks (1954)] and function of the foot during propulsion. Modelling PA kinematics during gait showed the PA to be acting quasi-isometrically or shortening throughout propulsion, despite concurrent MTP joint dorsiflexion (Caravaggi et al., 2010; Welte et al., 2021). Whilst the PA may return stored energy via recoil during the latter stages of gait, it is not mechanically possible to increase the magnitude of PA passive torques while the PA is shortening. Therefore, to increase torque about the MTP joint at this point in gait requires active muscular contributions, which our toe flexors appear well placed to provide. In the present study, we have shown that the foot's toe flexor muscles are structured to function at favourable positions for force generation as the MTP joint dorsiflexes through the propulsive phase. This provides further support to the notion that these muscles modulate forefoot stiffness for effective push-off more readily than passive structures. Moreover, the quasi-isometric function of the PA has been observed despite increasing gait speeds and greater mechanical demands placed upon the foot structure (Caravaggi et al., 2010; Farris et al., 2020). If forefoot stiffness were governed entirely by passive structures such as the PA, greater toe dorsiflexion would place greater strain on the elastic tissues; however, more forceful propulsions were not achieved with increased PA lengthening (Caravaggi et al., 2010), but via significantly greater PIM activation (Farris et al., 2019) without the need for increased MTP joint dorsiflexion (Farris et al., 2020). The effect of this activation on torque production is modulated by force–length–velocity states of the muscles involved, and our torque–angle relationships suggest muscle states will be favourable for converting activations to torque outputs. Therefore, our work advances a growing body of evidence that muscles acting in the foot are equally important, if not more so, as passive structures such as the PA in the generation of tension in the distal foot during the push-off phase in gait. The ability to push-off effectively is central to our ability to transition between walking step-cycles (Kuo, 2007; Kuo et al., 2005). As such, typical human bipedal gait requires suitably strong toe-flexor muscles.

However, the relatively low passive torques observed in our study at more neutral MTP joint angles may be in part explained by the fact that the arch of the foot remained relatively unloaded throughout the experiment, which may have limited the strain placed upon the PA. Tensioning of the foot's passive plantar structures *in vivo* appears to be initiated by the ankle plantar flexors pulling on the calcaneus and flattening the longitudinal arch during dynamic movements (Erdemir et al., 2004; Farris et al., 2020) rather than extension of the toes alone. Although ankle plantar flexors were activated when stimuli were applied to the PF tibial nerve site, it is unlikely that this loaded the PA as it would be under weight-bearing conditions. Thus, the passive torque–angle curve might shift leftward when the arch is more loaded. Indeed, Schuster et al. (2023) showed increased passive stiffness of the PA (and MTP joint) during toe extension when the load on the foot was greatly increased. This suggests the PA operated at longer lengths on a non-linear force–length curve when the foot was loaded. However, those authors used anaesthetic nerve blocks to prevent muscle activation. Without such techniques, passive stiffness under load is challenging to measure *in vivo* because arch loading activates the PIMs (Farris et al., 2019; Kelly et al., 2015). Therefore, we opted to quantify torque–angle curves in a minimally loaded foot.

### Implications for foot health

Our results have fundamental implications not only for our understanding of foot function but also for applied value. Strengthening foot muscles is considered a credible method to manage and mitigate age-related declines in mobility (Menz, 2015; Mickle et al., 2016a) and to increase aspects of physical performance; for example, horizontal jumping ability (Yamauchi and Koyama, 2019). Although prior studies have demonstrated a beneficial effect pertaining to toe flexor strength and hypertrophy, it is unclear to what extent clinically prescribed exercises aimed at activating PIMs such as short foot, toe scrunching and toe spreading (Lynn et al., 2012; Mickle et al., 2016a; Okamura et al., 2020; Ridge et al., 2017; Taddei et al., 2020) reflect improved dynamic function (Willemse et al., 2022). These exercises are performed with a predominantly neutral MTP joint and may not be encouraging training within the more optimal operating range of the muscles. We speculate that training the toe flexors throughout their functional operating range reflected by the torque–angle relationships may better strengthen the muscles and provide greater capacity to modulate MTP joint stiffness, potentially mitigating mobility issues associated with weaker or smaller PIMs (Mickle et al., 2016b).

### Limitations

A limitation of the current study is that we were not able to measure muscle fascicle length to ascertain force–length properties of individual PIMs. Therefore, we were unable to account for any *in vivo* series elasticity effects which cause shifts between torque–angle and force–length relationships (Brennan et al., 2017; Hoffman et al., 2012). Prior work suggests series elasticity influences PIM muscle–tendon dynamics (Kelly et al., 2019). Thus, future work should seek to assess force–length properties of toe flexor muscles and operating lengths during locomotion. However, many of the muscles involved have complex fibre arrangements, and existing *in vivo* ultrasound measurement methods cannot cope with non-planar and non-homogeneous fibre orientations. Computational musculoskeletal modelling and simulation may allow further progress in this area.

Furthermore, we employed peripheral nerve stimulation to evoke involuntary responses from the toe flexor muscles. A potential limitation of this method is that stimulating the tibial nerve at the PF may also inadvertently induce a small but detectable stimulation of the common peroneal nerve, as illustrated in Fig. S3. Therefore, it is plausible that slight activation of antagonistic muscles may have occurred in the PF conditions, resulting in a minor underestimation of the magnitude of torque contributions from the extrinsic foot muscles. Our use of supramaximal nerve stimulation also needs consideration. Activation of toe flexors during locomotor activities will predominantly be submaximal and the proportion of passive to active forces in a given position will probably shift toward passive forces. Submaximal conditions can also result in a rightward shift in the force–length relationship (Holt and Azizi, 2014).

Finally, it was not possible from our data to distinguish between individual synergistic intrinsic muscle contributions and determine whether one muscle has greater importance for the moment produced about the MTP joints than others. Similarly, we are unable to determine relative contributions from individual passive structures to passive torques. Further work aiming to partition the forces of the muscles and passive structures is required to precisely determine the active and passive force–length curves of each muscle that influence hallux and lesser digit flexion.

### Conclusion

In conclusion, we have quantified torque–angle relationships for active and passive tissues spanning the MTP joint in humans. In doing so, we provide valuable quantitative insight into the active and passive contributions to torque-generating capacity at the MTP joints. Both the plantar intrinsic and extrinsic muscle groups produced greater torque than a lumped combination of passive tissues. We also found that the ascending limb of the torque–angle relationships matched the range of motion where torque production is greatest during gait, suggesting torque production mechanisms are optimised during the push-off phase of walking. Future studies of foot mechanics might use the data presented here to help inform parameters for musculoskeletal models of the human foot that can be used to explore foot function further.

## Supplementary Material

10.1242/jexbio.249816_sup1 Supplementary information

Dataset 1. Individual torque-angle curve parameters and experimental measurements used to fit each respective curve. All active and passive torque-angle curve parameters and measurements for each experimental condition: HFT_MM_, HFT_PF_, LDFT_MM_ and LFDT_PF_. Active torque-angle measurements were recorded about the hallux and lesser digits via supramaximal tibial nerve stimulation at two stimulation sites: (a) the medial malleolus and (b) the popliteal fossa. Passive torque-angle measurements of the hallux and lesser digits were included to fit individual passive torque-angle relationships.
