# Supplementary material for: Torque–angle relationships of human toe flexor muscles highlight their capacity for propulsion in gait
Source: J Exp Biol. 2025 Jan 10;228(1):JEB249816. doi: 10.1242/jeb.249816 (PMC11744321; doi:10.1242/jeb.249816)
Supplement: Supplementary information [file jexbio-228-249816-s1.pdf]

Table S1. Summary of intrinsic and extrinsic toe flexor muscle physiological cross sectional area data (PCSA) from the literature, organised by muscle

Muscle	Species	Body mass (kg)	PCSA (cm^2)	PCSA/Mass$^{2/3}$	Source
Abductor Hallucis	Human	80*	9.9	0.53*	Tosovic et al. (2012)
	Human	80*	6.7	0.36*	Kura et al. (1997)
	Chimpanzee	54.6	5.4	0.37	Oishi et al. (2018)
	Bonobo	-	5.0	-	Oishi et al. (2018)
	Gorilla	118.5	7.6	0.31	Oishi et al. (2018)
	Orangutang	80	3.53	0.19	Oishi et al. (2018)
	Gibbon	6.3	0.8	0.23	Vereeke et al. (2005)
	Bonobo	60	4.29	0.28	Vereeke et al. (2005)
Flexor digitorum Brevis	Human	80*	5.3	0.29*	Tosovic et al. (2012)
	Human	80*	5.0	0.27*	Kura et al. (1997)
	Chimpanzee	54.6	3.4	0.24	Oishi et al. (2018)
	Bonobo	-	4.6	-	Oishi et al. (2018)
	Gorilla	118.5	7.7	0.32	Oishi et al. (2018)
	Orangutang	80	5.9	0.32	Oishi et al. (2018)
	Gibbon	6.3	0.25	0.07	Vereeke et al. (2005)
	Bonobo	60	1.36	0.09	Vereeke et al. (2005)
Flexor Hallucis Brevis	Human	80*	3.92	0.21*	Kura et al. (1997)
	Chimpanzee	54.6	4.9	0.34	Oishi et al. (2018)
	Bonobo	-	4.5		Oishi et al. (2018)
	Gorilla	118.5	11.0	0.46	Oishi et al. (2018)
	Orangutang	80	7.4	0.40	Oishi et al. (2018)
	Gibbon	6.3	-	0.20	Vereeke et al. (2005)
	Bonobo	60	3.06	0.21	Vereeke et al. (2005)
Flexor Hallucis Longus	Human	83	6.9	0.36	Ward et al. (2009)
Flexor Tibialis	Chimpanzee	72	6.7	0.39	Holowka & O'Neill (2013)
	Gibbon	6.3	1.92	0.56	Vereeke et al. (2005)
	Bonobo	60	6.22	0.41	Vereeke et al. (2005)
Flexor Digitorum Longus	Human	83	4.4	0.23	Ward et al. (2009)
Flexor Fibularis	Chimpanzee	72	15.1	0.87	Holowka & O'Neill (2013)
	Gibbon	6.3	4.67	1.37	Vereeke et al. (2005)
	Bonobo	60	14.3	0.93	Vereeke et al. (2005)

*indicates that no body mass data were provided in the paper for the cadaver specimens. To allow a calculation of PCSA isometrically scaled to body mass, the approximate average body mass of male & females in Australia and USA (countries in which studies were conducted) of the reported specimen age range (60-80 year old males and females: Tosovic et al (2012); 50-90 years old males and females: Kura et al. (1997)) was used (80kg). Sources of body mass data – Fryer et al. (2018). Mean Body Weight, Height, Waist Circumference, and Body Mass Index Among Adults: United States, 1999–2000 Through 2015–2016. *National Health Statistics Report* (122); Australian Bureau of statistics – 4338.0 – Profiles of Health, Australia, 2011-13, https://www.abs.gov.au/ausstats/abs@.nsf/lookup/4338.0main+features212011-13)

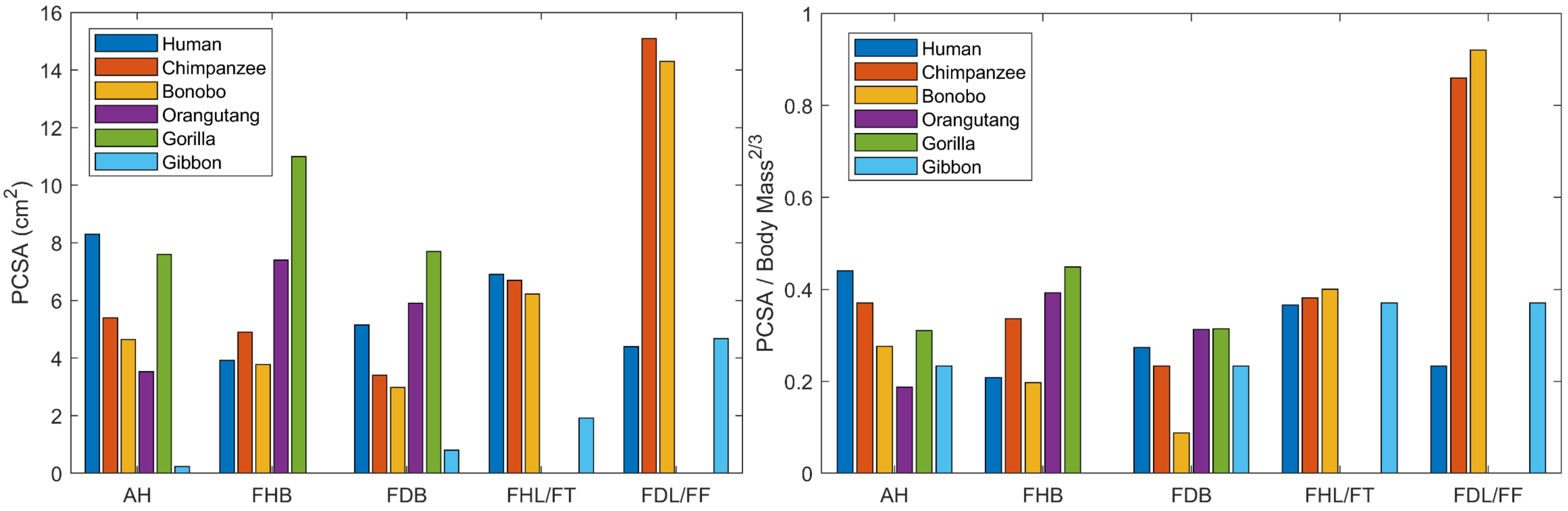

Fig. S1. **Comparison of toe flexor muscle sizes among humans and other extant ape species in terms of raw PCSA (left panel) and isometrically scaled PCSA (right panel).** Abductor Hallucis (AH), Flexor Hallucis Brevis (FHB), Flexor Digitorum Brevis (FDB), Flexor Hallucis Longus (FHL) or Flexor Tibialis (FT), and Flexor Digitorum Longus (FDL) or Flexor Fibularis (FF). Although not identical in terms of muscle attachments, the FT and FF from studies of Chimpanzees, bonobos, and gibbons were compared to FHL and FDL, respectively in humans as the most similar.

References

Tosovic D, et al. (2012). The architecture and contraction time of intrinsic foot muscles. *Journal of Electromyography and Kinesiology*, 22, 930–938; Kura H, et al. (1997) Quantitative analysis of the intrinsic muscles of the foot. *The Anatomical Record: An Official Publication of the American Association of Anatomists* 249.1: 143-151; Oishi, M, et al. (2018). Multivariate analysis of variations in intrinsic foot musculature among hominoids. *Journal of anatomy*, 232.5: 812-823. Vereecke, E, et al. (2005). Functional analysis of the foot and ankle myology of gibbons and bonobos. *Journal of Anatomy*. 206, 453–476; Ward, SR, et al. (2009). Are current measurements of lower extremity muscle architecture accurate?. *Clinical orthopaedics and related research* 467.4: 1074-1082. Holowka, N.B. & O'Neill, M.C. (2013). Three-dimensional moment arms and architecture of chimpanzee (Pan troglodytes) leg musculature. *Journal of Anatomy* 223.6: 610-628

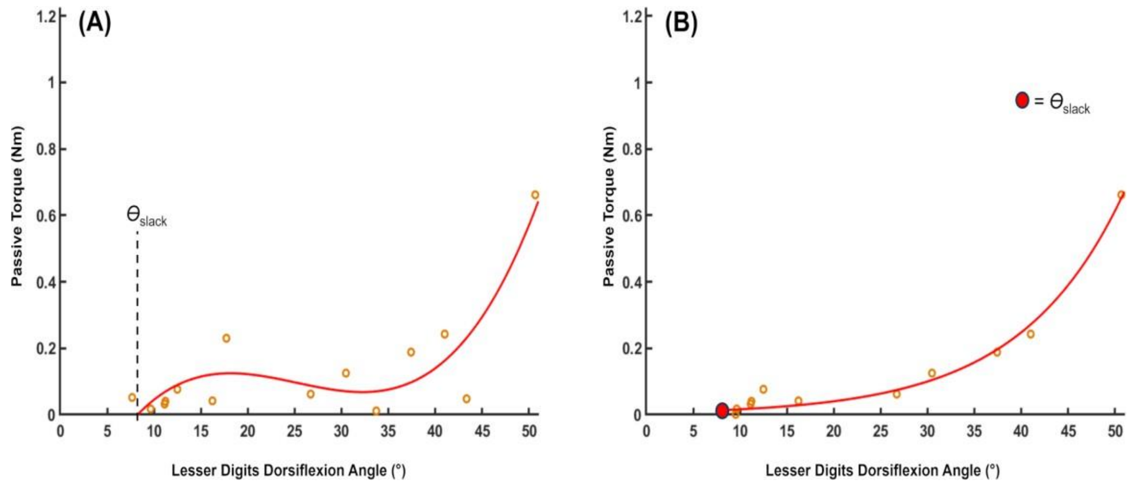

Fig. S2. Curve fitting process to determine passive torque-angle relationship. Panel A displays a third-order polynomial curve fit to resting passive torque data to determine slack angle (ϴslack), defined as the dorsiflexion angle corresponding to the curve intersection at 0 Nm (dashed black line). An exponential function (B) was then fit to passive torque-angle data beyond Θ_{slack}: $T_{passive} = Ae^{k(\theta - \theta slack)}$, with Θ denoting the modelled lesser digit MTP joint angle and exponential coefficients, *k*, the stiffness of the curve representing passive stiffness of the MTP joint(s), and *A*, its curvature. It should be noted that this passive data includes contributions from all passive structures, particularly the plantar aponeurosis and therefore is not reflective of the passive force from the muscles involved. The data presented within the figure is taken from participant 8 (n = 1).

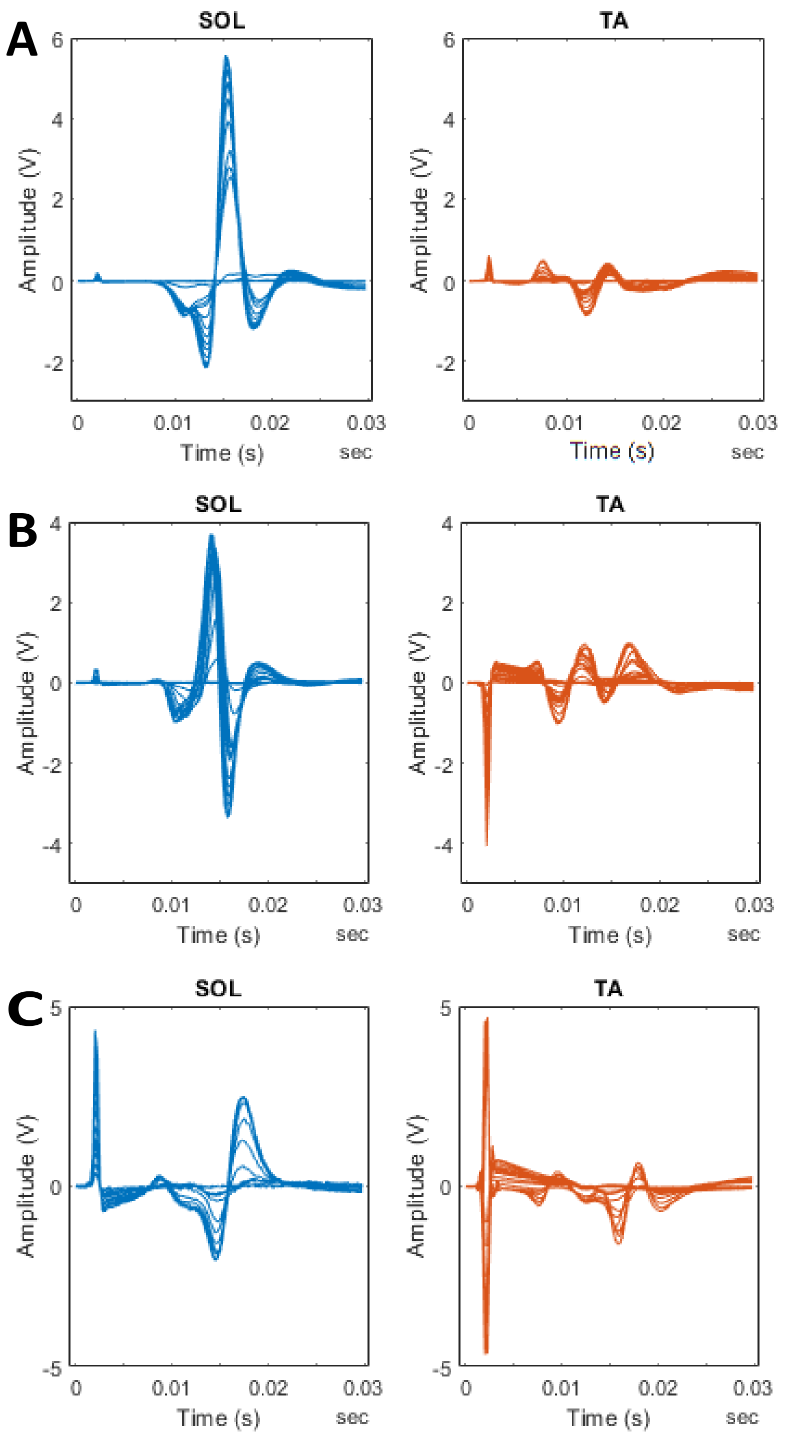

Fig. S3. **Surface EMG recordings from soleus (SOL – blue) and tibialis anterior (TA – orange) in response to peripheral tibial nerve stimulation at the popliteal fossa.** EMG recordings from SOL and TA are presented as surrogates showing tibial nerve stimulation and any incidental common peroneal nerve stimulation, respectively. Recordings were taken from three participants (A- C). The m-wave response can be observed ~0.01-0.025 s following stimulus delivery at 0 s.

Dataset 1. Individual torque-angle curve parameters and experimental measurements used to fit each respective curve. All active and passive torque-angle curve parameters and measurements for each experimental condition: HFT_{MM}, HFT_{PF}, $LDFT_{MM}$ and $LFDT_{PF}$. Active torque-angle measurements were recorded about the hallux and lesser digits via supramaximal tibial nerve stimulation at two stimulation sites: (a) the medial malleolus and (b) the popliteal fossa. Passive torque-angle measurements of the hallux and lesser digits were included to fit individual passive torque-angle relationships.

Available for download at
https://journals.biologists.com/jeb/article-lookup/doi/10.1242/jeb.249816#supplementary-data

Journal of Experimental Biology • Supplementary information
